# Novel TRPV1 Channel Agonists With Faster and More Potent Analgesic Properties Than Capsaicin

**DOI:** 10.3389/fphar.2020.01040

**Published:** 2020-07-14

**Authors:** Yorley Duarte, Javier Cáceres, Romina V. Sepúlveda, Diego Arriagada, Pedro Olivares, Ignacio Díaz-Franulic, Jimmy Stehberg, Fernando González-Nilo

**Affiliations:** ^1^Center for Bioinformatics and Integrative Biology, Facultad de Ciencias de la Vida, Universidad Andres Bello, Santiago, Chile; ^2^Laboratorio de Neurobiologia, Instituto de Ciencias Biomédicas, Facultad de Medicina y Facultad de Ciencias de la Vida, Universidad Andres Bello, Santiago, Chile; ^3^Centro Interdisciplinario de Neurociencia de Valparaíso, Universidad de Valparaíso, Valparaíso, Chile

**Keywords:** transient receptor potential vanilloid 1 channels, TRPV1, drug discovery, capsaicin, analgesic, carrageenan, allodynia, von frey

## Abstract

The transient receptor potential vanilloid 1 (TRPV1) ion channel is a member of the family of Transient Receptor Potential (TRP) channels that acts as a molecular detector of noxious signals in primary sensory neurons. Activated by capsaicin, heat, voltage and protons, it is also well known for its desensitization, which led to the medical use of topically applied TRPV1 agonist capsaicin for its long-lasting analgesic effects. Here we report three novel small molecules, which were identified using a Structure-Based Virtual Screening for TRPV1 from the ZINC database. The three compounds were tested using electrophysiological assays, which confirmed their capsaicin-like agonist activity. von Frey filaments were used to measure the analgesic effects of the compounds applied topically on tactile allodynia induced by intra-plantar carrageenan. All compounds had anti-nociceptive activity, but two of them showed faster and longer lasting analgesic effects than capsaicin. The present results suggest that TRPV1 agonists different from capsaicin could be used to develop topical analgesics with faster onset and more potent effects.

## Introduction

Transient receptor potential (TRP) ion channel superfamily involves 30 members, organized in seven subfamilies, from which it is possible to distinguish ten thermosensitive TRP (ThermoTRP) channels (Latorre et al., 2009; Diaz-Franulic et al., 2016). Among the ThermoTRP channels, the transient receptor potential vanilloid 1 (TRPV1) is particularly interesting since it behaves as a polymodal receptor activated by heat, protons, voltage, lipids, and vanilloid ligands such as capsaicin, the active component of “hot chili peppers” (Owsianik et al., 2006; Baez-Nieto et al., 2011). The TRPV1 channel is expressed in sensory neurons of the dorsal root and trigeminal ganglia, and its activation is critical for pain responses (Caterina et al., 1997; Caterina et al., 2000). Paradoxically, despite the fact that TRPV1 channel activation triggers pain sensation in several pathological conditions (Geppetti et al., 1988), the topical application of the TRPV1 channel agonist capsaicin has analgesic effects, resulting from the functional desensitization of primary nociceptors (Jancso et al., 1967; Szolcsanyi, 2004). This desensitization process occurs after sustained stimulation, rendering the channel unresponsive to further stimulation (Koplas et al., 1997; Numazaki et al., 2003).

The structure of TRPV1 channel obtained by Cryogenic electron microscopy (cryo-EM) revealed the molecular architecture of the channel (Cao et al., 2013; Liao et al., 2013b), allowing for computational studies of channel gating, pharmacology, and ion permeation (Darre and Domene, 2015; Darre et al., 2015; Poblete et al., 2015; Chugunov et al., 2016). The available TRPV1 channel structure allows the search for small molecules with potentially high affinity for the capsaicin binding site by performing a virtual screening (VS) over large libraries containing millions of compounds. Such strategy avoids the expensive and time-consuming high throughput screening of thousands of molecules using *in vitro* assays. This approach has become crucial in drug discovery, with encouraging results for the identification of early hits and lead compounds (Chin et al., 2004; Liu et al., 2007; Nagarajan et al., 2012). Here we performed structure-based virtual screening (SBVS) to identify novel TRPV1 agonists using a commercially available library of 112,935 compounds from the ZINC database (http://zinc.docking.org/), specifically using the Diversity 3 catalog. Diversity 3 is a freely accessible database administered by the Developmental Therapeutics Program of the National Cancer Institute, NCI. After filtering by physicochemical and pharmacokinetic properties of putative TRPV1 agonists, we selected and tested *in vitro* three of the compounds using electrophysiological assays, all exhibiting a half-maximal response (EC_50_) 4–9-fold lower than capsaicin. Behavioral tests performed in Sprague–Dawley rats using tactile allodynia from inflammatory pain showed that all compounds possessed analgesic activity comparable to capsaicin. Interestingly, two of the compounds induced analgesic effects that were more potent, longer lasting analgesic effects and had an earlier onset than capsaicin. This study opens the possibility of developing novel and more effective pain relief compounds targeting the TRPV1 channel.

## Materials and Methods

### Computational Methods

#### Protein Receptor and Small Ligand Preparation

In the computational pipeline implemented for the discovery of new TRPV1 agonists, we required the 3D structure of both the TRPV1 channel and every small ligand tested *in silico* in the present study. For TRPV1, the structure of the channel resolved by cryo-EM in complex with resiniferatoxin (RTX) and double-knot toxin (DkTx) was selected as the receptor [PDB ID: 3J5Q] (Milne et al., 2013). The structure of the TRPV1–RTx–DkTx complex was retrieved from the Protein Data Bank [www.rscb.org] in.pdb format, which contains the atomic coordinates for each residue of the protein. The protein was prepared using AutoDockTools by adding the missing hydrogens to the structure, computing the partial charges for each atom and producing a united atom representation of the macromolecule. In the structure, only the polar hydrogens are displayed as explicit atoms (*i.e.*, OH), and the non-polar ones are represented by adding their partial charges to the closest heavy atom (*i.e.*, CH_3_) (Perryman et al., 2014). The receptor was stored in AutoDock pdbqt format where ‘pdbqt’ stands for protein data bank (pdb) format file plus charges (q) and AutoDock atom types (t). The vanilloid site was visually inspected to identify the most relevant residues described in the literature for vanilloid agonist binding to determine the conformational space where the search takes place (grid). Using the AutoDock Vina software (Trott and Olson, 2010), the grid center was calculated by obtaining the three-dimensional coordinates for the *α*-carbons of the residues Y511, M547, T550, E570, and L669 of the monomer D of the channel and by calculating the arithmetic mean of their coordinates. The search space was defined to cover the whole capsaicin binding pocket, resulting in a grid with dimensions 20 Å × 20 Å × 30 Å among the X, Y, and Z axis respectively. Using the AutodockVina software, a test run was performed with default docking parameters to observe whether the docking results converge within the binding site. The small molecules tested were obtained from the ZINC database (Irwin and Shoichet, 2005) in mol2 format, which comprises an all-atom representation of the molecule as well as the atomic partial charges and the atomic bonds order. They were prepared using the toolkit OpenBabel, which performs the same steps described above for the receptor and additionally, includes the rotatable bonds as active torsions, indicating the conformational degrees of freedom for the molecule. The protonation states for the small ligands were maintained as in the 2D representation available in the ZINC database (Irwin and Shoichet, 2005), which corresponded to the most likely state at pH 7.0, predicted using LigPrep (LigPrep). Every small molecule was stored in a pdbqt format as described above.

### Virtual Screening

The molecules tested in the VS correspond to a subset of the ZINC (NCI Diversity 3 catalog) (Irwin and Shoichet, 2005). The docking simulations were performed using the AutoDock Vina software (Trott and Olson, 2010) with the docking parameters (Perryman et al., 2014) and the conformational search space (grid) previously described. The results were ranked using their predicted affinity, and the best 1,000 molecules were used for the refinement of the compound selection. Due to the number of molecules to be docked, the script was set to use multiple cores simultaneously, in order to optimize the docking process and organize the results. The obtained binding energy (kcal/mol), which indicates the potential strength with which a ligand could bind to the TRPV1 receptor, was calculated based on the scoring function used by the AutoDock Vina program, using a binding energy assessment to assign the best conformation. The scoring function includes a sum of intramolecular and intermolecular contributions, which include van der Waals forces, hydrogen bonds, desolvation, electrostatic energies, and the energy of rotatable bonds between heavy atoms in the ligand (Lim et al., 2011). Among all interactions occurring in the active site, the ligand–receptor electrostatic interactions are the most significant because they allow assigning the putative strength of binding and the position of the ligand in the active site. In this study, the selection of molecules was done using a minimal docking energy of −7.0 kcal/mol, which is the *in silico* binding energy of capsaicin with TRPV1.

### *In Vitro* Evaluation of TRPV1 Channel Modulators

#### Molecular Biology

Salts were obtained from Sigma-Aldrich. *In vitro* synthesis of TRPV1 cRNA was carried out with the Message Machine kit (Ambion) according to the manufacturer’s instructions. *Xenopus laevis* care, surgery, and oocyte preparation are described in detail elsewhere (González-Pérez et al., 2008).

#### Electrophysiological Recordings

For two electrode voltage clamping‎ (TEVC), oocytes were impaled with two 3M KCl-filled capillary Ag/AgCl electrodes with resistances in the 0.2–1.0 MΩ range (Gonzalez-Perez et al., 2010). Current recordings were performed using an OC-725C amplifier (Warner Instruments) through a PCI-6035 interface (National Instruments) under the command of pCLAMP software (Molecular Devices). Normal recording solution consisted of (in mM) 100 NaCl, 2.5 KCl, 1.8 CaCl_2_, 1 MgCl_2_, and 10 HEPES-NaOH (pH 7.4). TRPV1 channel activation was determined by measuring the current amplitude elicited by a depolarizing voltage pulse to 30 mV before and after the addition of the compound to the external solution. The maximal channel activation was tested by adding a saturating concentration of capsaicin (50 µM), and channel activation at each concentration was normalized by the maximal TRPV1 response in the presence of capsaicin. Five independent experiments were performed for each compound and averages are expressed with their respective Standard Error (SE).

### *In Vivo* Testing of Analgesic Effects for TRPV1 Channel Modulators

Male Sprague–Dawley rats (approximately 200–300 g body weight) were used. Food and water were supplied *ad libitum* and the animals were kept in a 12 h light–dark cycle in their home cages throughout the study. All procedures were in accordance with the U.S. National Institutes of Health guidelines and were approved by the bioethical Committee of the Universidad Andres Bello, Acta 11/2016.

#### Reagents and Compound Preparation

2% *λ*-Carrageenan (Santa Cruz Biotechnology) was administered subcutaneously to induce tactile allodynia according to previous studies (Yoshimura and Yonehara, 2001; Fehrenbacher et al., 2012). Capsaicin was obtained from Sigma. Compounds 1, 2, and 3 were obtained from the National Cancer Institute. Capsaicin and compounds 1, 2, and 3 were used at EC_50_, 10-fold EC_50_, 100-fold EC_50_, 1,000-fold EC_50_ concentrations based on their respective EC_50_ values obtained using electrophysiological recordings *in vitro* (see above). Novo base moisturizing cream (Charles Lasserre) was used as vehicle for all tested compounds including capsaicin and was used alone as vehicle control. The different concentrations used can be found in **Table 3**.

#### Groups

Rats were randomly assigned into the following groups for all compounds: EC_50_, 10-fold EC_50_, 100-fold EC_50_, 1,000-fold EC_50_, a control group treated with cream only and a control group for 2% *λ*-Carrageenan in which saline was injected instead of *λ*-Carrageenan.

#### Acute Inflammation Model

The *λ*-Carrageenan model for inflammatory pain was used to induce mechanical allodynia lasting for less than 24 h, as reported elsewhere (Yoshimura and Yonehara, 2001; Fehrenbacher et al., 2012). In short, animals were injected with 100 µl of 2% *λ*-Carrageenan diluted in saline, or saline alone as vehicle control, into the left hind paw. One hour later, capsaicin or one of the three compounds dissolved in cream was administered on the plantar surface until fully absorbed using a cotton swab. Then the rat was placed in the test chamber and acclimated for 5 min.

#### Mechanical Stimuli

The day before the experiments, tactile thresholds were measured in all animals to attain a baseline and to ensure normal sensitivity to tactile stimuli. On the day of the experiment, the cream that contained the compounds was applied only once, 5 min before the evaluation began, and the effects of each compound was measured over time, once per hour, for eight consecutive hours. Von Frey filaments were used to measure plantar mechanical sensitivity and inflammatory allodynia. Briefly, the rats were placed into the arena with a mesh floor and after the 5-min habituation, were tested using von Frey filaments. The following von Frey filaments were applied against the plantar surface: 52.2 g (512 mN), 26.1 g (256 mN), 13.0 g (128 mN), 6.52 g (64 mN), 3.26 g (32 mN). To measure tactile response, the up-down method was used (Dixon, 1980), with slight modifications: the starting filament (13.0 g) was applied perpendicularly to the plantar surface of the hind paw until it buckled for 3 s or until the rat withdrew its foot. If the rat responded to the filament, the procedure was repeated with the smaller filament (6.52 g) and then repeated sequentially until the smallest filament that elicits a response was identified. If the starting filament did not elicit a response, larger filaments were evaluated sequentially until the smallest filament capable of eliciting a response was identified. To confirm that the identified filament was the smallest that could elicit a response, the filament was changed to the next smaller filament with no response and then increased again to the filament that produced a response. The responses (X) and lack of responses (0) were recorded to obtain the tabular values from Dixon (1980), corresponding to:

**Table:** 

Pattern	K value	mN	Grams
00x0x	−0.439	512	52.2
0x0x	−0.500	256	26.1
x0x	−0.842	128	13.0
xx0x	−0.890	64	6.52

#### Dose–Response Curve and Statistical Analysis

Data from von Frey filaments was obtained as a dose**–**response curve for each compound over the course of 8 h. All groups were tested for normality and a two-way ANOVA with Bonferroni *post hoc* test was used to analyze differences. Differences were considered significant when p < 0.05 and averages are shown ± standard error of the mean (SE).

Data was analyzed in terms of the response to each compound compared to vehicle and to capsaicin, and shown as fold change.

The paw withdrawal threshold (PWT) for each time point was calculated by using the following equation proposed by Dixon, 1980:

$$\text{PWT}=50\%\;\text{threshold}\;(\text{g})=(10^{\left[\text{Xf}+\text{K}\delta \right]})/104^{4})$$

where Xf = value of the smallest filament capable of eliciting a response (in log units),

K = tabular value
δ = mean difference between stimuli (in log units)

To estimate the percentage of analgesia, the filament values (in g) obtained from the vehicle control (cream) group were defined as 0% of the effect of a drug and the filament values (in g) of the saline-injected control were defined as the maximum value that the effect of the drugs could reach (100%). The saline injection was considered the correct control to define the values for 100% analgesia rather than baseline for each animal despite the fact that the injection of saline produced a transient increase in tactile allodynia. The percentage of analgesia was estimated as percentage of the effect of each compound from the analgesia range between the cream and saline controls for that particular time point.

The ED_50_ (median effective dose) is defined as the dose in which 50% of the animals show effects of treatment. It was calculated using the least-squares method (Ishikawa et al., 2014), following the following formula:

% MPE=100×(PWT-minimum possible value)/(maximum possible value−minimum possible value)

Percentage values were established between 0 and 100 (for our data) that correspond to the maximum possible effect (MPE) of a compound. These values were plotted linearly, in relation to the concentrations of compounds used. The value of 50% on the Y axis corresponds to the ED_50_, and thus a linear regression was performed, and the Y value = 50% was interpolated to obtain the ED_50_ concentration. Given that the compounds showed different kinetics, an ED_50_ for each time point was calculated and compared. The time of the maximal analgesia was used to determine the ED_50_ at maximal analgesia.

## Results

### Novel Putative Agonists of the TRPV1 Channel Revealed by Virtual Screening

**Figure 1:**
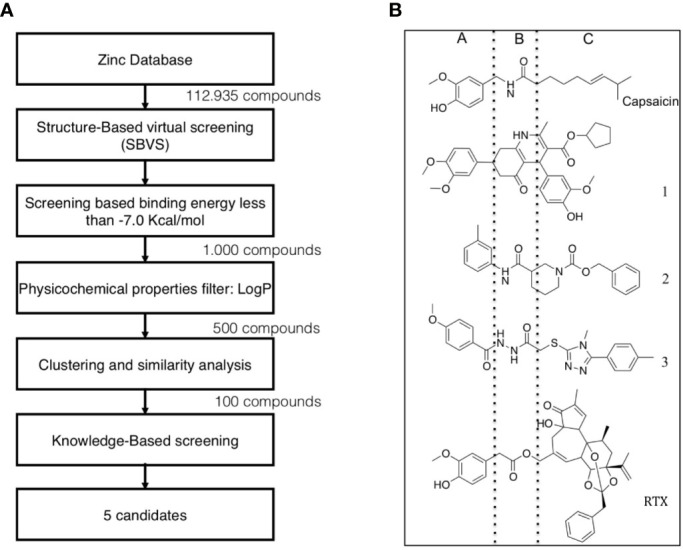
Workflow of TRPV1 Virtual Screening. **(A)** A SBVS over a ZINC Database with 112,935 compounds was performed using the TRPV1 channel structure as target. Results were filtered by excluding all molecules with binding energy below −7 kcal/mol, which reduced the candidate molecules to 1,000. A restriction by LogP values compatible with the ADME criteria was subsequently applied, followed by clustering and bibliography-based selection, which rendered five potential candidate molecules. **(B)** Structural comparison between capsaicin, the novel TRPV1 channel agonists found in this study and Resiniferatoxin. The molecules were decomposed into three regions, called A, B, and C. A-region comprises the Vanilloid ring, B-Region comprises a linker containing the electron donor–acceptor pair and C-region represents the hydrophobic region of each molecule.

The vanilloid binding site in the structure of the open TRPV1 channel in complex with RTX and DkTx (PDB ID: 3J5Q) was used as the target for a SBVS (Liao et al., 2013b). **Figure 1A** describes the workflow of VS step by step. We screened the NCI Diversity 3 library from the ZINC database consisting of 112,935 molecules. The VS was performed with AutoDockVina (Trott and Olson, 2010; Perryman et al., 2014) confining the docking grid around the transmembrane segments 2–3 that contains most of the molecular determinants of TRPV1 vanilloid sensitivity (Jordt and Julius, 2002; Gavva et al., 2004; Yang et al., 2015). All compounds were ranked according to their docking binding energy, and the associated value to capsaicin was settled as the value cutoff (−7 kcal/mol), thus reducing the number of candidate molecules to 1,000. The SwissADME web tool (Daina et al., 2017) was used to predict the Absorption, Distribution, Metabolism, and Excretion (ADME) properties of these 1,000 compounds, and the octanol/water partition coefficient (LogP value < 5) was used to rank the compounds and reduce our candidate molecules to 500. We performed a clustering analysis that considers chemical features such as the presence of a vanilloid ring at one molecule end, an electron acceptor group at the middle region, and a highly hydrophobic region, equivalent to the n-carbon acyl chain present in capsaicinoids (**Figure 1B**). This step reduced the candidate compounds to 100. In a final step of the VS, a visual analysis of each molecule and all potential binding sites within the grid using molecular docking was performed. The only three compounds that showed physical proximity to the amino acids known to modulate the TRPV1 vanilloid sensitivity were selected and considered putative TRPV1 channel agonists (**Table 1**). A full description of the proposed putative molecular interactions obtained from the docking assays is reported as **Supplementary Material** and discussed in the *Discussion* section.

**Table 1:** Theoretical parameters obtained *in silico* for each compound.

Compound	Number	Structure	EA Kcal/mol	LogP	EC_50_ (nM)
Capsaicin		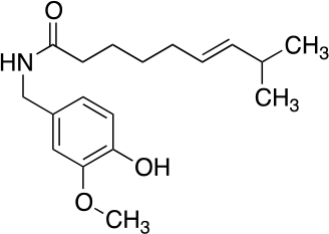	-7.0	3.58	440 ± 66
Compound	1	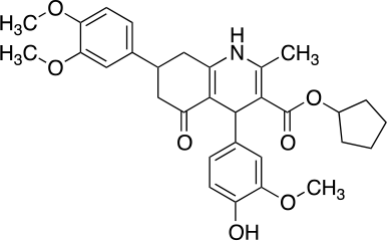	-9.3	4.65	53 ± 6
Compound	2	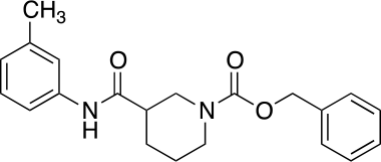	-9.2	3.31	53 ± 4.3
Compound	3	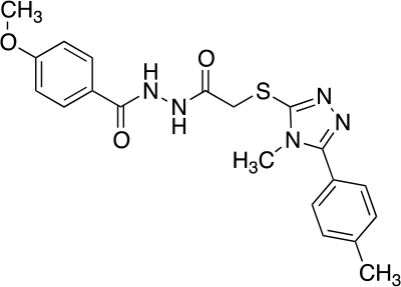	-9.1	2.78	92 ± 10
Resiniferatoxin		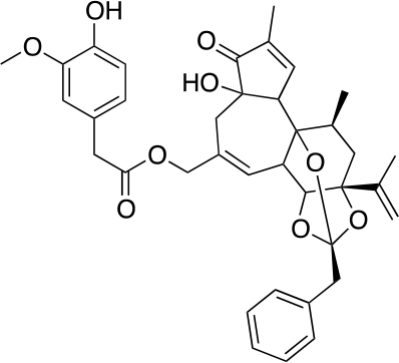	-9.0	4.51	0.7 ± 3

Structure (column 3), potential binding energy (column 4), theoretical Octanol/water partition coefficient (LogP; column 5) and empirical EC_50_ obtained using the two electrode voltage clamp assays (column 6) are shown for capsaicin, compounds 1, 2, 3 and Resiniferatoxin (RTX). Values are expressed as average ± standard error (SE).

### *In Vitro* Validation of Putative TRPV1 Channel Agonists

**Figure 2:**
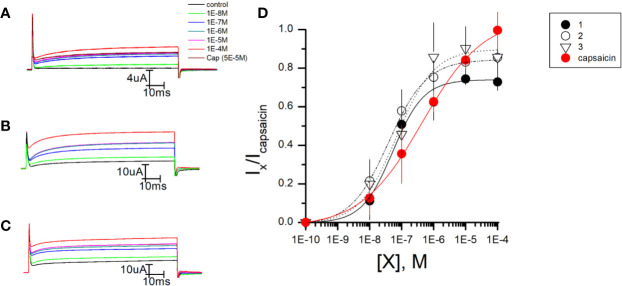
*In vitro* evaluation of putative TRPV1 agonists. **(A)** Representative experiments of TEVC recordings in Xenopus Oocytes expressing TRPV1 channels during a +30 mV pulse at increasing concentrations of compounds 1 **(A)**, 2 **(B)**, and 3 **(C)**. **(D)** Dose–response curve of putative TRPV1 agonists. Steady-state currents elicited by a voltage pulse at +30 mV in the presence of compound 1 (filled circles), compound 2 (empty circles), compound 3 (inverted triangles), and capsaicin (red circles) normalized by the currents elicited in the presence of capsaicin (I_x_/I_capsaicin_) as a function of capsaicin or test compound concentration (X). The data was fitted using Hill Equation: $\frac{Ix}{lcapsaicin}=Imax*{\left[X\right]}^{n}/\left({EC}_{50}^{n}+{\left[X\right]}^{n}\right)$. The following parameters were estimated for compounds 1, 2, 3, and capsaicin: EC_50_ of 53 ± 6 nM, 53 ± 4.3 nM, 92 ± 10 nM, and 440 ± 66 nM respectively; Hill coefficients (n) of 0.73, 0.9, 1.06, and 0.45 respectively and maximal effect was 0.8, 0.73, 0.89, and 1, respectively.

To test the validity of *in silico* predictions, we tested the effects of the three compounds as putative agonists of the TRPV1 channel by measuring their effect on channel activity using the two-electrode voltage clamp (TEVC) technique. We recorded the TRPV1 channel currents after applying a depolarizing voltage step to +30 mV in control conditions and after increasing the concentration of the test compound in the bath. This current was plotted relative to the current evoked by the full agonist capsaicin at a saturating concentration. **Figures 2A–C** show representative current recordings for each compound and in **Figure 2D**, the dose**–**response relationship for the different compounds in a semi-logarithmic scale. These results are summarized in **Table 1**, together with the calculated EC_50_. All tested compounds showed to be TRPV1 channel agonists. Compounds 1, 2, and 3 exhibited an EC_50_ ± S.E of 53 ± 6 nM (p < 0.005), 53 ± 4.3 nM (p < 0.005), and 92 ± 10 nM (p < 0.005), respectively. The EC_50_ of all compounds was between 4 and 9-fold lower than capsaicin (440 ± 66 nM), and these differences were statistically significant after performing unpaired student t-test when comparing each compound with capsaicin. The three compounds required lower concentrations to reach saturation (hence, their lower EC_50_), but were unable to match the maximal effect of capsaicin at higher concentrations.

### *In Silico* ADME Prediction

Many potential drug candidates fail to reach clinical use because of their unfavorable ADME parameters (absorption, distribution, metabolism, and excretion), increasing the costs of new drug development (Hop, 2012). Therefore, early assessment of the pharmacokinetic properties of potential therapeutic agents is an essential step in the drug development process that can guide optimization efforts towards improved compounds. Therefore, we performed a computational study of the screened compounds together with capsaicin and RTX for the assessment of ADME properties. The values obtained are depicted in **Table 2**. Polar surface area (TPSA), number of hydrogen bond donors (n-OH, NH), number of hydrogen bond acceptors (n-ON), some pharmacokinetics, and drug-likeness properties were calculated using SwissADME web tool (Daina et al., 2017). A correlation between physicochemical properties and successful drug development can be examined by comparing the structural features of orally administered drugs and drug candidates, as introduced by the Lipinski rules (Lipinski et al., 1997). From all the parameters obtained, the three compounds satisfy Lipinski’s rules; LogP values <5, number of HBD (donor) <5, and number of HBA (acceptor) is <10. The molecular weight of compounds 2 and 3 was <500 g/mol, thus predicting high absorption, transportation, and diffusion. The molecular weight of molecule 1 exceeded the 500 value (533.61 g/mol), as shown in **Table 2**. The three compounds presented LogP values as well as gastrointestinal absorption (GI) that met the permeation requirements of an orally administered compound, showing high bioavailability scores. The three compounds showed suitable values to be skin permeable. Moreover, synthetic accessibility scores to lab-scale were found to be below 5 for compounds 2 and 3, indicating they can be easily synthesized on a large scale, but compound 1 showed a high synthetic complexity. The three compounds followed the criteria for orally active drugs and skin permeability, and therefore, their putative analgesic effects were tested using *in vivo* assays.

**Table 2:** *In silico* predictions of ADME, pharmacokinetics, physicochemical properties, and Lipinski parameters for each compound.

Property	Capsaicin	1	2	3	RTx
MW	305.41	533.61	352.43	411.48	626.74
TPSA	58.56	103.32	58.64	123.44	100.52
n-OH,NH	3	7	3	5	8
n-ON	2	2	1	2	1
Bioavailability score	0.55	0.55	0.55	0.55	0.55
Synthetic accessibility	2.32	5.29	3.22	3.31	7.67
Skin permeation	-5.62	-6.25	-6.10	-6.84	-6.41
GI absorption	High	High	High	High	High
Druglikeness	yes	yes	yes	yes	yes

Different predicted properties (column 1) are shown for capsaicin (column 2), compound 1 (column 3), compound 2 (column 4), compound 3 (column 5), and Resiniferatoxin (RTX; column 6). TPSA, Topological polar surface area; MW, Molecular weight; n-OH,NH, Number of hydrogen bond donors; n-ON, Number of hydrogen bond acceptors.

### *In Vivo* Testing of the Novel Compounds

**Figure 3:**
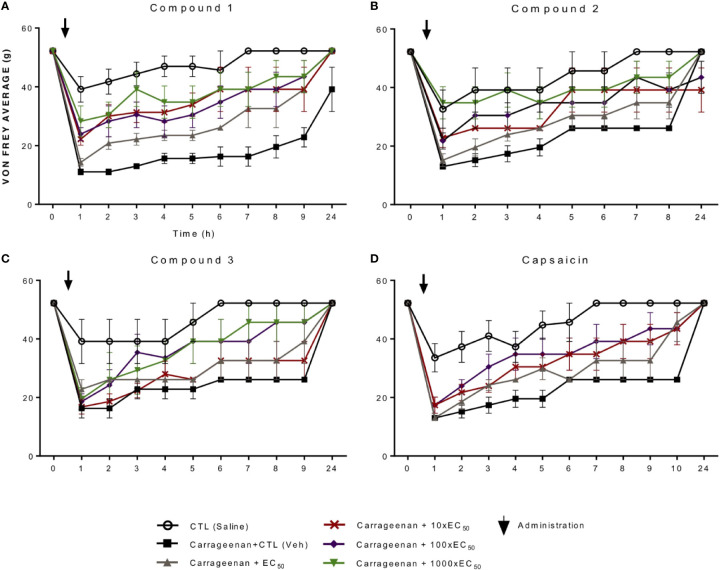
Effects of compounds on tactile allodynia produced by carrageenan-induced inflammatory pain in rats. The effects on tactile thresholds (g) using von Frey filaments before (0) and at different times (1–9, 24 h) after the subcutaneous injection (arrow) of saline (open circles) or carrageenan followed by application of vehicle (cream alone; filled squares), or together with increasing concentrations equivalent to the EC_50_ (gray triangle), 10xEC_50_ (red cross), 100xEC_50_ (purple rhomboid) and 1,000xEC_50_ (green inverted triangle) of compound 1 **(A)**, compound 2 **(B)**, compound 3 **(C)**, and capsaicin **(D)**. Statistics are shown in **Table 4**.

We used the carrageenan model of inflammatory pain to compare the analgesic effects of the different molecules to that of capsaicin. In this model, 2% carrageenan was injected subcutaneously in the plantar base of the hind paw and either capsaicin or the experimental molecules, dissolved in a topic base, were applied at different concentrations onto the plantar surface only once. To be able to compare the analgesic effects of the compounds to capsaicin, the EC_50_ obtained *in vitro* (see above) and multiples of 10, 100, and 1,000 of the EC_50_ (written as EC_50_, 10-fold, 100-fold or 1,000-fold) for each compound were compared. The corresponding concentrations are reported in **Table 3**. The effects over time of each compound on the mechanical allodynia induced by subcutaneous carrageenan were measured using von Frey filaments. Two controls were used; base cream as vehicle control (control) and a saline injection to control for carrageenan-induced allodynia (saline). The analgesic effect of compound 1 is shown in **Figure 3A**, of compound 2 in **Figure 3B**, and compound 3 in **Figure 3C**, and of capsaicin in **Figure 3D**. All compounds induced dose dependent analgesia, which increased over time. Given that each compound was applied only once (after time 0) at four different concentrations and tested over time on carrageenan-induced allodynia, several statistical analyzes were performed. **Table 4** summarizes the results shown in **Figure 3**. Compound 1 showed significant analgesic effects compared to vehicle (cream) at 10xEC_50_ 1 h after application, effect that lasted for 8 h. At higher concentrations, it showed significant analgesic effects 1-h post application, effect that lasted at least for 9 h. Compound 2 showed significant analgesic effects at 100xEC_50_ 2 h post application. At higher concentrations, compound 2 induced significant analgesia from the first hour post application, effect that lasted for 3 h. Compound 3 showed significant effects at concentrations x100 and 1,000x EC_50_, effects that were significant only between 7- and 9-h post application. Capsaicin had significant analgesic effects only at the 100xEC_50_ concentration, effects that were significant between 3- and 5-h post application (see **Table 4**).

**Table 3:** Concentrations of compounds used for the *in vivo* studies.

	Molecule 1 (nM)	Molecule 2 (nM)	Molecule 3 (nM)	Capsaicin (nM)
**EC_50_**	53 ± 6	53.0 ± 4.3	92 ± 10	440 ± 66
**10xEC50**	530	530	920	4400
**100xEC50**	5300	5300	9200	44000
**1000xEC50**	53000	53000	92000	N/A

All concentrations used were multipliers of the EC_50_ concentration found in vitro for each compound. The multipliers are shown in column 1 (EC_50_, 10xEC_50_, 100xEC_50_, 1000xEC_50_) and their corresponding concentrations are shown for molecule 1 (column 2), compound 2 (column 3), compound 3 (column 4), and capsaicin (column 5).

**Table 4:** Statistical analysis of tactile thresholds obtained using von Frey filaments.

	Compound 1				Compound 2				Compound 3				Capsaicin		
Time (h) /EC50	x1	x10	x100	x1000	x1	x10	x100	x1000	x1	x10	x100	x1000	x1	x10	x100
0	–	–	–	–	–	–	–		–	–	–	–	–	–	–
1	–	*	*	**	–	–	–	***	–	–	–	–	–	–	–
2	–	****	**	***	–	–	*	**	–	–	–	–	–	–	–
3	–	****	**	****	–	–	–	***	–	–	–	–	–	–	*
4	–	***	*	***	–	–	*	*	–	–	–	–	–	–	*
5	–	****	**	***	–	–	–	–	–	–	–	–	–	–	*
6	–	**	**	***	–	–	–	–	–	–	–	–	–	–	–
7	–	**	***	***	–	–	**	**	–	–	–	*	–	–	–
8	–	*	**	***	–	–	–	**	–	–	*	*	–	–	–
9	–	–	**	**	–	–	–	–	–	–	*	*	–	–	**
24	–	–	–	–	–	–	–	–	–	–	–	–	–	–	–

Analgesic effects of the compounds relative to vehicle. The different time points are shown in column 1, concentrations (as multipliers of EC50) are shown for each compound (compound 1, columns 2-5; compound 2, columns 6-9; compound 3; columns 10-13 and capsaicin, columns 14-16). Statistical differences were analyzed using 2-way ANOVA and Bonferroni post-hoc test; -, is non-significant; *p < 0.05; **p < 0.01; ***p < 0.001; ****p < 0.0001.

Based only on significant effects compared to the cream, compound 1 had the earliest long-lasting analgesic effects at the lowest concentration, followed by compound 2, then capsaicin and finally compound 3.

**Figure 4:**
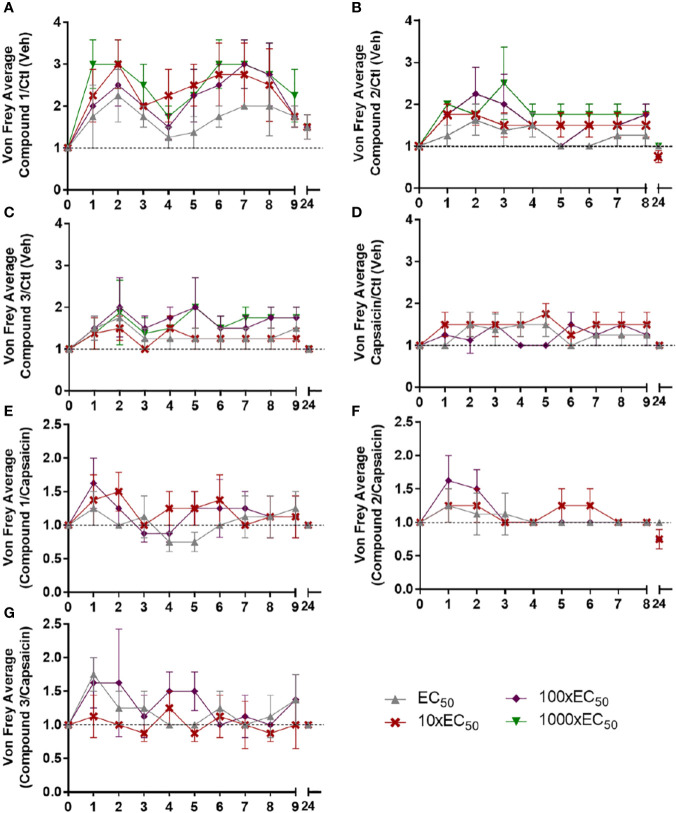
Ratio of tactile thresholds between the different compounds and vehicle or capsaicin. The results of tactile thresholds for compound 1 **(A)**, compound 2 **(B)**, compound 3 **(C)**, and capsaicin **(D)** were divided by the thresholds obtained for the vehicle group. The results of tactile thresholds for compound 1 **(E)**, compound 2 **(F)**, compound 3 **(G)** were divided by the thresholds obtained for the capsaicin group. Note that compounds 1 and 2 show effects at earlier time points compared to capsaicin. Concentrations: EC_50_ (gray circle, 10xEC_50_ (red cross), 100XEC_50_ (purple rhomboid), 1000xEC_50_ (green inverted triangle).

As can be observed in **Figures 4A–D**, the ratios between compound and control (base cream) values suggest different patterns of analgesia over time for each compound, with novel compounds 1 and 2 having greater effects at shorter times than capsaicin (**Figures 4A, B** compared to **D**). This greater analgesia at earlier times is more clearly observed when plotting the ratio between the compounds and capsaicin (**Figures 4E–G**).

**Figure 5:**
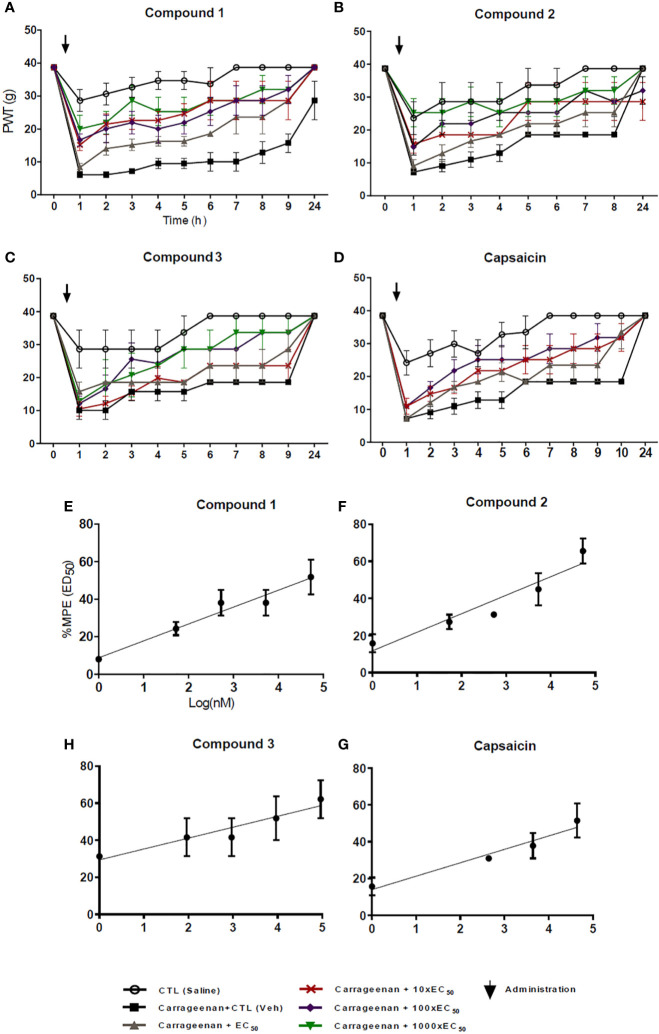
Paw withdrawal thresholds (PWT) and maximal possible effect (%MPE) to extrapolate the median effective dose (ED50). The PWT was estimated for compound 1 **(A)**, compound 2 **(B)**, compound 3 **(C)**, and capsaicin **(D)**. Concentrations: EC_50_ (gray circle, 10xEC_50_ (red cross), 100XEC_50_ (purple rhomboid), 1,000xEC_50_ (green inverted triangle). The ED_50_ was extrapolated at 50% from the linear fit of the %MPE values at the log10 of each concentration for compound 1 **(E)**, compound 2 **(F)**, compound 3 **(G)**, and capsaicin **(H)**.

The effects of the novel compounds and that of capsaicin are compared by estimating their median effective dose (ED_50_), which is defined as the dose in which 50% of the animals show effects of treatment. To estimate the ED_50_, it was first necessary to estimate the paw withdrawal threshold (PWT) for each time point and each concentration, using the von Frey filament results shown in **Figure 4** and tabular values published by Dixon (Dixon, 1980) (See *Materials and Methods*). The PWT for each compound is shown in **Figures 5A–D**.

The range of analgesia extends between the saline injection (negative control lacking carrageenan) and the effect of the vehicle (cream). Thus, the analgesia effect of each compound was calculated as a percentage from the difference between both controls at each time point for each concentration. Thus, using the PWT values, the ED_50_ was extrapolated from the linear fit (**Figures 5E–H**).

The overall parameters obtained for each compound are shown in **Table 5**. Summarizing, capsaicin showed an ED_50_ of 86.9 µM, a maximal analgesia of 86%, which was obtained 4 h after the application of 44 µM capsaicin. The analgesic effects of capsaicin were first significant 3 h after application and lasted 2 h. Compound 1 had an estimated ED_50_ of 37.7 µM, a maximal analgesia of 83%, which was obtained 3 h after the application of 53 µM. The first significant analgesic effect appeared 1 h after application and analgesia lasted for 8 h.

**Table 5:** Summary of results.

Summary Results				
Compound	1	2	3	Capsaicin
ED50 of maximal effect	37.7 µM	6.8 µM	139.3 µM	86.9 µM
Maximal percentage of analgesia attained	83%At 3 h	100%at 3 h	75%at 7 h	86%At 4 h
Concentration at maximal analgesia	53 µM	53 µM	92 µM	44 µM
Time to first effect	Hour 1	Hour 1	Hour 7	Hour 3
Effect duration (h)	8	3	2	2

Estimated ED_50_ at maximal effect (row1), maximal analgesia attained (row 2), concentration at maximal analgesia (row 3), time to first significant effect (row 4) and effect duration (row 5) are shown for compound 1 (column 2), compound 2 (column 3), compound 3 (column 4) and capsaicin (column 5).

Compound 2 showed an ED_50_ of 6.8 µM, maximal analgesia reached 100%, 3 h post-application at a 53 µM concentration. First significant analgesia was found 1 h post-application and lasted for 3 h.

Finally, compound 3 showed an ED_50_ of 139.3 µM, a maximal analgesia of 75%, which was obtained 7 h after the application of 92 µM. The first significant analgesia was found 7 h post-application and lasted 2 h.

In summary, compounds 1 and 2 show the fastest analgesic effects, followed by capsaicin, and then by compound 3. Compounds 1 and 2 showed the lowest ED_50_, followed by capsaicin and compound 3. Compound 1 had the longest lasting effect; 8 h. Compounds 1, 2, and capsaicin reached similar levels of maximal analgesia (range between 83 and 100%) despite having different kinetics. Compounds 1 and 2 showed a lower time to effect onset and were faster to reach maximal analgesia than capsaicin. Compound 3 showed the lowest parameters among all compounds tested.

## Discussion

Here, we first used computational methods to identify novel TRPV1 channel ligands among a ZINC database containing hundreds of thousands of small molecules, from which three were identified, and their effectiveness was demonstrated by *in vitro* and *in vivo* assays. The approach of Structure Based Virtual Screening is an essential tool in aiding fast and efficient drug discovery, mostly because it considers not only the chemical similarity among ligands and candidate compounds but also takes into account the three-dimensional structure of the target (Lionta et al., 2014). A similar theoretical-experimental strategy has been used to find several lead molecules that have emerged as compounds with therapeutic potential for different molecular targets (Jenkins et al., 2003; Xing et al., 2011). Our study followed the standard procedure for VS, which is to select the binding site and library, computational docking, binding energy-based classification, and Lipinski’s rule selection. We also added a visual inspection step to increase the chances to find TRPV1 ligands. *In vitro* measurements of TRPV1 activity in response to the compounds showed that all three novel compounds had agonist effects similar to those found for capsaicin. However, they showed an EC_50_ four to nine times lower than capsaicin.

The use of VS to search for novel pain-relief compounds targeting the TRPV1 channel has been reported previously for the identification of novel TRPV1 channel antagonists. Using a variety of pharmacophores as structural templates, Goldmann and colleagues reported 12 TRPV1 channel antagonists with binding affinities in the nM range after performing VS over the LifeChem library containing 305,841 compounds (Goldmann et al., 2015). Due to their *in vivo* effectiveness in blocking TRPV1 channels (Tafesse et al., 2014; Voight et al., 2014), these compounds could be considered as promising candidates to become novel pain relief compounds. We followed a different approach, as we performed the VS directly on the capsaicin-binding site of the TRPV1 channel instead of using an existing pharmacophore (Gavva et al., 2004; Liao et al., 2013a). After checking *in vitro* that the selected ligands were TRPV1 channel agonists, we moved on to *in vivo* assays to evaluate their analgesic effects. The results from the *in vitro* assays shown here suggest that the novel compounds have agonist effects on the TRPV1 channel but are no proof that the ligands are actually binding to the vanilloid-binding site of the TRPV1 channel. Our *in silico* results from molecular docking between the compounds and the TRPV1 channel suggest that the three novel compounds could be binding to the same vanilloid-binding site as capsaicin (see **Supplementary Figure 1**). However, site mutations and agonist competition assays are necessary to ascertain whether the novel compounds share their mechanisms of action with that of capsaicin and whether they induce desensitization of the TRPV1 channel as a mechanism for their analgesic effects. Further experiments are necessary to identify and characterize in detail their mechanisms of action.

The *in vivo* experiments using the inflammatory pain model showed that each compound has a different pattern of analgesia over time. Additionally, two of the novel compounds (compounds 1 and 2) showed faster and more long-lasting analgesic effects than capsaicin, which are attained within the first hour of application. The maximal analgesic effect reached by each compound was similar to that obtained by capsaicin. Hence, compounds 1 and 2 are good candidates for chemical optimization to generate novel analgesic compounds with faster and longer-lasting effects than capsaicin, requiring lower concentrations to attain similar analgesic effects. The requirement of lower doses may lead to lesser side effects, such as burning, stinging, and erythema (Sawynok, 2005), although this issue needs to be validated empirically. From the theoretical point of view, the estimated solubility (LogP; **Table 1**) and skin permeation (**Table 2**) obtained *in silico* are roughly similar in all compounds, including capsaicin. However, the possibility that empirical differences in solubility of the compounds in the cream or in their skin permeability could contribute to the differences in the analgesic effects and their kinetics cannot be ruled out.

Capsaicin has been widely used to induce pain, but the mechanisms associated with its analgesic effects are not fully understood [for a review see (Fattori et al., 2016)]. Studies evaluating capsaicin in inflammatory pain models in rodents are scarce and have mostly used intraplantar injections rather than topic application (Menendez et al., 2004; Baamonde et al., 2005). This is particularly interesting, as many clinical studies in humans have been performed using topic administration of capsaicin in different pain syndromes (reviewed in (Burness and Mccormack, 2016)). To our knowledge this is the first study which assesses the effects of topically applied capsaicin in tactile allodynia using a rat model of inflammatory pain. Moreover, we also describe the analgesic effects obtained over time of three novel compounds with TRPV1 agonist activity and analgesic effects. Two of these compounds show faster and longer lasting effects than capsaicin when applied topically and could be used as a basis for the development of novel drugs to treat chronic pain.

## Conclusions

We applied the structure-based virtual screening method using as a target the vanilloid pocket binding of TRPV1, through the NCI Diversity 3 library of compounds to identify novel TRPV1 agonists. Three of these compounds were experimentally tested in electrophysiological assays; all acted as TRPV1 agonists, exhibiting an EC50 4–9-fold lower than capsaicin. After the *in vitro* validation of these three compounds as TRPV1 channel agonists, we moved forward into an *in vivo* evaluation of their analgesic effects in a rat model of inflammatory pain. The *in vivo* results showed that compounds 1 and 2 possess faster and longer-lasting analgesic effects than capsaicin. Both compounds open new opportunities to be chemically optimized or be used as a reference for further drug discovery to find new agonists of TRPV1 as pain relief drugs.

## Data Availability Statement

The raw data supporting the conclusions of this article will be made available by the authors, without undue reservation, to any qualified researcher.

## Ethics Statement

The animal study was reviewed and approved by bioethical Committee of the Universidad Andres Bello, Acta 11/2016.

## Author Contributions

YD, JC and RS conceived and analyzed the *in silico* experiments. YD prepared and wrote the original draft. ID-F conducted the *in vitro* experiments. JS, PO and DA conducted the *in vivo* experiments. FG-N and JS reviewed and edited the manuscript.

## Funding

This project was supported by the FONDECYT grants 1170733 (FG-N) and Nº1200452 (JS), PMI UAB1301, the US Air Force Office of Scientific Research (AFOSR) under Award FA9550-16-1-0384 (FG-N), FONDECYT Postdoctorado 3170599 (ID-F) and the US Army, Rational Design of Pain Inhibitors: Target TRPV1 Channel: W911NF-14-1-0520 (FG-N). The Centro Interdisciplinario de Neurociencia de Valparaiso is a Millennium Institute supported by the ICM-ANID, PROYECTO (P09-022-F), CINV.

## Conflict of Interest

The authors declare that the research was conducted in the absence of any commercial or financial relationships that could be construed as a potential conflict of interest.

## Supplementary Material

Supplementary Figure 1 Molecular Docking of putative TRPV1 channel agonists. The figure depicts the most probable conformation and possible binding mode for each putative agonist at the vanilloid binding site of TRPV1. a) Capsaicin (orange) can establish several putative bonds and interactions with the channel. Its A-region could form a π–π stacking with Y511 (yellow line); the B-Region could produce two hydrogen bonds with T550 (red lines), and the C-Region could produce hydrophobic interactions with M547 and L669 (green line). b) Compound 1 (violet), has an A-region in close proximity to Y511, but no interaction is predicted, while the B-Region could form three hydrophobic interactions with T550 (green line), and the C-Region could share two hydrogen bonds with M547 and F543 (red line). c) Compound 2 (green) shows a possible interaction between its A-region and Y511 (green line); the C-Region could have a hydrophobic interaction with L669 (green line) and a π–π stacking with residue F543 (yellow line). d) Compound 3 (cyan) has an A-region in close proximity to Y511, but no interaction is predicted, while its B-Region could establish two hydrogen bonds with T550 (red line), and its C-Region could establish a double π–π stacking with residue F543 (yellow line).

**Supplementary Material**

**Potential protein–compound interactions at the vanilloid binding site of the TRPV1 channel**

Based on molecular docking and experiential studies published elsewhere, at a molecular level, the TRPV1–capsaicin complex exhibits three potentially crucial interactions. The first one is at the extracellular region of the binding pocket, where the residues M547 and L669 interact with the carbon acyl chain of capsaicin. A second one is found in the middle region where the amide group of capsaicin interacts through hydrogen bonds with the residues L669 and T550. Finally, in the intracellular region of the binding pocket, a *π*–*π* stacking interaction between Y511 and the vanilloid ring is found (**Supplementary Figure 1a**). These theoretical interactions are in agreement with both experimental and theoretical studies involving capsaicin binding to TRPV1 channels (Darre and Domene, 2015; Poblete et al., 2015; Yang et al., 2015). The molecular docking analysis of the three novel compounds shows an adopted tail up–head down conformation (Tail for the C section and head for the A portion of each molecule; **Supplementary Figure 1b****)** similar to the configuration taken by capsaicin inside the vanilloid binding pocket (**Supplementary Figures 1b–d**). In compounds 1 and 3 (as seen in **Supplementary Figure 1b**), the methoxy-phenol and triazole rings are pointed toward M547 and L669, establishing a hydrophobic interaction (**Supplementary Figures b, d**). Additionally, the hydroxyl group of methoxy phenolic moiety from compound 1 can form a hydrogen bond with the carbonyl oxygen of F543 (top red broken line). In contrast, compounds 2 and 3 form a well-established π–π stacking interaction with the aromatic ring of the same residue. An additional, π–π stacking interaction could take place between the triazole ring of compound 3 and F543 (broken yellow line). Different from compounds 1 and 3, the carbonyl oxygen of the ester group from compound 2 points towards M547, increasing the distance between them to >4Å.

The amide linker of compound **3** can form two hydrogen bonds with the carbonyl oxygen and the amine group of T550 (**Supplementary Figure 1d**), which is absent in molecules 1 and 2. The vanilloid ring of the three compounds lies close to the lower end of the fourth transmembrane segment (S4), where a π–π stacking interaction is likely to occur with Y511 due to their closeness. Unlike the hydrophobic carbon chain in the capsaicin molecule, in compounds 1, 2, and 3 there is a substituted phenyl moiety in the hydrophobic side chain, which could be important for improving potency, presumably related to optimal π–π stacking and hydrophobic interactions with M547 and F543 residues. Moreover, the stability of the novel compounds could be putatively coordinated by the pi-pi interactions with F543, which is not observed in TRPV1 structures incorporating RTX (3J5Q) and Capsaicin (3J5R) systems. This specific interaction could increase the affinity to S3 and S4 segments, confine the ligand to that interaction zone and reduce the direct interactions with the S5-S6 segment residues involved in channel gating. Empirical data is required to evaluate to which extent these *in silico* predictions are empirically involved in the effects of the compounds on TRPV1 channels.
