# Supplementary figures and images for: Novel TRPV1 Channel Agonists With Faster and More Potent Analgesic Properties Than Capsaicin
Source: Front Pharmacol. 2020 Jul 14;11:1040. doi: 10.3389/fphar.2020.01040 (PMC7372189; doi:10.3389/fphar.2020.01040)

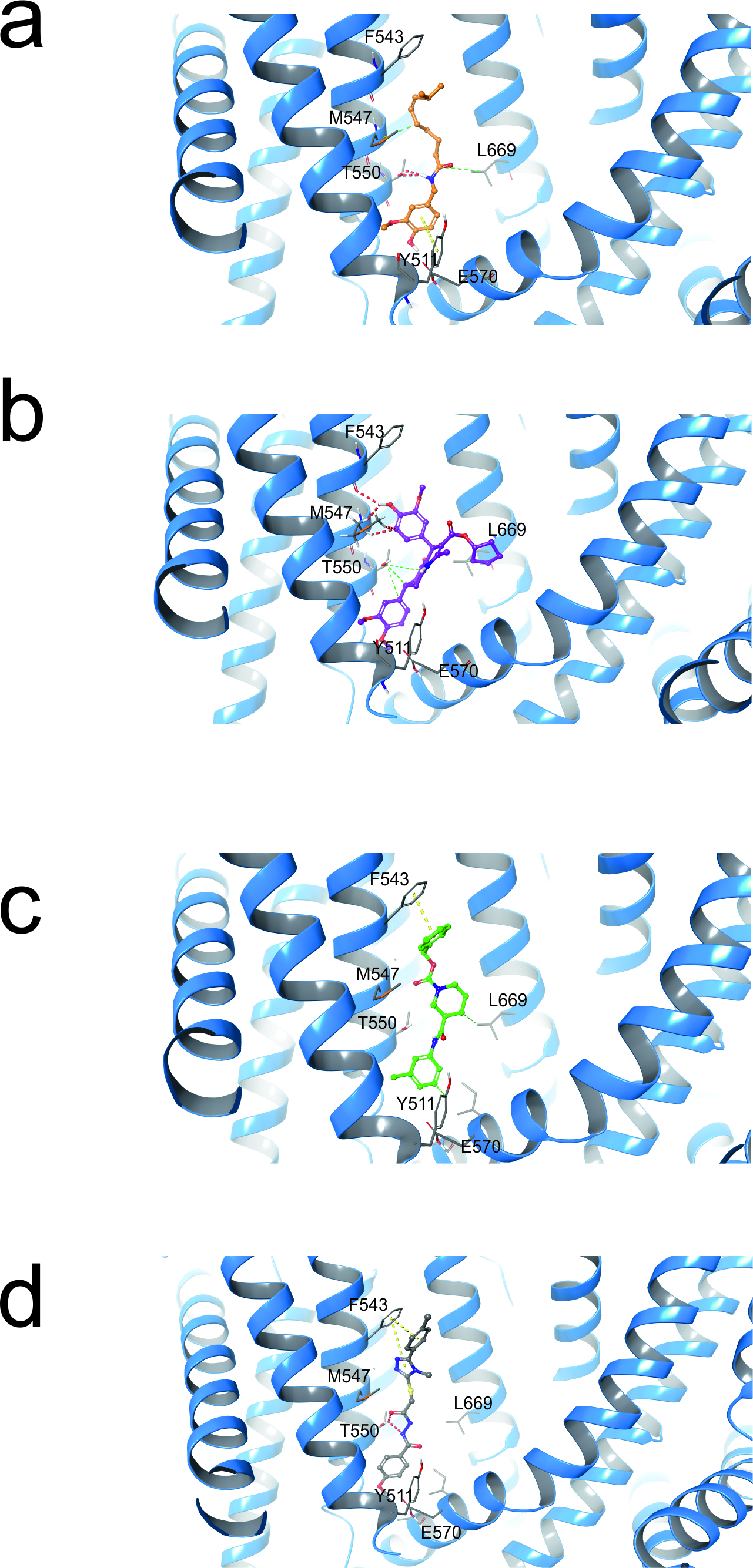

Supplement: Supplementary Figure 1 — Molecular Docking of putative TRPV1 channel agonists. The figure depicts the most probable conformation and possible binding mode for each putative agonist at the vanilloid binding site of TRPV1. a) Capsaicin (orange) can establish several putative bonds and interactions with the channel. Its A-region could form a π–π stacking with Y511 (yellow line); the B-Region could produce two hydrogen bonds with T550 (red lines), and the C-Region could produce hydrophobic interactions with M547 and L669 (green line). b) Compound 1 (violet), has an A-region in close proximity to Y511, but no interaction is predicted, while the B-Region could form three hydrophobic interactions with T550 (green line), and the C-Region could share two hydrogen bonds with M547 and F543 (red line). c) Compound 2 (green) shows a possible interaction between its A-region and Y511 (green line); the C-Region could have a hydrophobic interaction with L669 (green line) and a π–π stacking with residue F543 (yellow line). d) Compound 3 (cyan) has an A-region in close proximity to Y511, but no interaction is predicted, while its B-Region could establish two hydrogen bonds with T550 (red line), and its C-Region could establish a double π–π stacking with residue F543 (yellow line). [file Image_1.jpeg]
